# Chemical interactions and dynamics with femtosecond X-ray spectroscopy and the role of X-ray free-electron lasers

**DOI:** 10.1098/rsta.2017.0464

**Published:** 2019-04-01

**Authors:** Philippe Wernet

**Affiliations:** Institute for Methods and Instrumentation for Synchrotron Radiation Research, Helmholtz-Zentrum Berlin für Materialien und Energie GmbH, 12489 Berlin, Germany

**Keywords:** X-ray spectroscopy, metal complexes, metalloproteins, X-ray free-electron laser, photochemical reactions, excited-state dynamics

## Abstract

X-ray free-electron lasers with intense, tuneable and short-pulse X-ray radiation are transformative tools for the investigation of transition-metal complexes and metalloproteins. This becomes apparent in particular when combining the experimental observables from X-ray spectroscopy with modern theoretical tools for calculations of electronic structures and X-ray spectra from first principles. The combination gives new insights into how charge and spin densities change in chemical reactions and how they determine reactivity. This is demonstrated for the investigations of structural dynamics with metal K-edge absorption spectroscopy, spin states in excited-state dynamics with metal 3p-3d exchange interactions, the frontier-orbital interactions in dissociation and substitution reactions with metal-specific X-ray spectroscopy, and studies of metal oxidation states with femtosecond pulses for ‘probe-before-destroy’ spectroscopy. The role of X-ray free-electron lasers is addressed with thoughts about how they enable ‘bringing back together’ different aspects of the same problem and this is thought to go beyond a conventional review paper where these aspects are formulated in italic font type in a prequel, an interlude and in a sequel.

This article is part of the theme issue ‘Measurement of ultrafast electronic and structural dynamics with X-rays'.

**Prequel**. *In the end, I could not hold myself back. Originally I wanted to write a more ‘normal’ review on the* *methods and applications of time-resolved X-ray spectroscopy using X-ray free-electron lasers in the area of liquid-phase photochemistry. But I decided not to do this. Instead I am* *trying to do something different and this is an attempt to explain what it is. The idea may be unusual but hopefully will turn out to be useful. It is motivated by the request to keep this contribution accessible to non-specialists combined with my ambition to make it attractive to those already established in the field. Instead of giving a complete account following a chronological order or following a structure imposed by methods or systems, I tried to assign classical and modern studies to topics of enduring relevance such that each collection of studies spans from fundamentals to future prospects. With this completely subjective and necessarily incomplete selection, which of course is nothing else than re-sorting a very limited set of representative investigations, I want to at least help motivating future applications of time-resolved X-ray spectroscopy in photochemistry. In fact, I have another intention. When put together, such as here, these collections, hopefully, expose a common pattern: ‘things are coming together’. X-ray free-electron lasers may be considered transformative tools that give access to new observables which, when combined with established knowledge, can be used to bring back together different aspects of the same problem. Aspects that belong together but got separated with time or increasing specialization in the various subfields, aspects that were treated in different disciplines or communities, aspects that seemed unrelated or remained separated because they contradicted each other or seemed inconsistent with one another, now, with the advent of X-ray free-electron lasers, come together. The seemingly inexplicable inconsistencies and contradictions vanish and new insight is enabled. With a sequel at the end of this contribution I will try to illustrate and validate this claim based on a famous historic example*.

## 1. Time-resolved X-ray spectroscopy of photochemical reactions of 3d transition-metal complexes

This contribution deals with time-resolved femtosecond X-ray spectroscopy of photochemical reactions in solution at X-ray free-electron lasers (XFELs). It is complemented by the contribution in the same theme issue by Leone *et al.* on time-resolved X-ray spectroscopy using table-top high-order harmonic generation sources with applications to the photophysics of gas-phase and solid-state samples. Furthermore, closely related aspects of the ultrafast dynamics of nuclei and electrons in molecules and proteins and how these can be probed with X-ray spectroscopic and scattering methods are treated in other contributions in this theme issue. The spirit of my contribution is inspired by the introduction to this theme issue by Jon Marangos where original or historic publications are related to recent studies with XFELs.

A number of reviews of photochemical reaction dynamics were published last year as part of a special issue on ‘Ultrafast Processes in Chemistry’ in *Chemical Reviews* edited by Thomas Elsaesser [1], and the contributions by Chergui & Collet, and by Ponseca *et al.* explicitly address the use of X-ray methods for probing structural dynamics in photochemical reactions [2,3]. In addition, numerous aspects of the present contribution are covered to some extent in the books *X-ray Free Electron Lasers—Applications in Material, Chemistry and Biology* edited by Bergmann *et al.* [4], and *Synchrotron Light Sources and Free-Electron Lasers—Accelerator Physics, Instrumentation and Science Applications* edited by Jaeschke *et al.* [5]. They all build the basis for the present contribution. By no means can my contribution, and in fact it is not intended to, give a complete account of the literature on time-resolved X-ray spectroscopy of photochemical reactions with XFELs and the reader is referred to the references above for an overview of the field.

Here I focus on time-resolved X-ray spectroscopic studies of 3d transition-metal complexes. 3d transition-metal systems are essential in many photochemical processes from photocatalytic solar-fuel production [6,7] to photosynthetic water splitting with metalloproteins [8,9]. A mechanistic understanding at the molecular level of photocatalytic and photochemical processes of optically excited 3d transition-metal systems may help in finding new ways to predict and control photochemical reactivity and selectivity. In turn, such understanding of the fundamental chemistry could ultimately help making available new sources of energy.

X-ray spectroscopy gives direct access to the electronic structure locally at the probed site due to the elemental specificity of atomic core-level energies. The specificity to the probed metal site by tuning the incident photon energy to an absorption edge of the metal turns out to be essential for all the studies discussed here. It gives direct access to the ‘delocalized valence electrons that participate in bonding’ (quotation from the introduction to this theme issue by Jon Marangos). It is one aim of this contribution to show how X-ray spectroscopy at XFELs enables new insights into the metal-ligand bonds in 3d transition-metal complexes. Of particular interest are methods probing the 3d transition-metal L and K absorption edges at photon energies of around 1–10 keV because they give a metal-centric view of the electronic structure at the reactive metal site. In order to illustrate how time-resolved X-ray spectroscopy complements other time-resolved spectroscopic and scattering methods, this contribution therefore also details to some extent and independently of the use of XFELs the information content of 3d transition-metal L- and K-edge spectroscopy. The importance of novel theoretical *ab initio* methods for effective interpretation of the experimental observables is highlighted.

Compared to table-top and synchrotron radiation X-ray sources, XFELs offer unprecedented peak brilliance at photon energies of up to 10 keV and beyond (see the contribution in this theme issue by Schoenlein *et al.*). This contribution focuses primarily on time-resolved X-ray absorption spectroscopy (XAS), X-ray emission spectroscopy (XES) and resonant inelastic X-ray scattering (RIXS). While XAS of the light elements C, N and O in small molecules is addressed in the introduction by Jon Marangos, it is one purpose of this contribution to introduce the reader to XAS of 3d transition-metal systems. RIXS will be highlighted as a way to access the low-energy excitations from meV to eV thereby giving access to ligand-field and charge-transfer excitations in 3d transition-metal systems. The book chapter on ‘Resonant Inelastic X-ray Scattering (RIXS) Studies in Chemistry: Present and Future’ by Marcus Lundberg and myself further details this aspect of studying 3d transition-metal systems with RIXS [10].

XAS, XES and RIXS uniquely benefit from the tuneable, intense and well-collimated radiation from XFELs and they are well suited to probe the electronic structure of 3d transition-metal systems in solution or in operando conditions. Where deemed insightful, time-resolved X-ray spectroscopy studies of photochemical reactions in the gas phase including X-ray photoelectron spectroscopy (XPS) are briefly addressed here. This will also allow introducing time-resolved electron spectroscopy for chemical analysis (ESCA) where ESCA is one of the most widely used methods to determine the chemical state of a material. It is noteworthy that this contribution also includes a discussion of studies making use of femtosecond X-ray pulses from XFELs that may be less obvious or less common but that is considered very important: For the study of X-ray sensitive solution samples such as high-valent metalloproteins and metal complexes in physiological or in operando conditions, femtosecond X-ray pulses from XFELs enable probing the radiation-sensitive system before it is altered or destroyed by X-ray induced sample damage. This ‘probe-before-destroy’ spectroscopic approach directly derives from the ‘diffract-before-destroy’ concept for imaging matter that Jon Marangos addresses in his introduction to this theme issue.

Areas that are not covered here include time-resolved X-ray probing of 4d and 5d metal systems [2] and of materials for solar energy conversion [2,3], time-resolved X-ray spectroscopy of small molecules and organic systems in solution probing C, N, O and S K-edges [11–13], surface chemical reactions [14–16], femto-, pico- and nanosecond time-resolved X-ray spectroscopy at synchrotron radiation X-ray sources [2,3,17–24], and time-resolved X-ray scattering of photochemical reactions in solution [25–31] and in the gas phase [32,33].

The content of this contribution, still focusing on the ultrafast dynamics of 3d transition-metal systems, is effectively motivated by two questions: Why using X-rays and why using XFELs? The aim is to give answers by discussing four selected topics: structural dynamics with metal K-edge absorption spectroscopy, spin states in excited-state dynamics with metal 3p-3d exchange interactions, frontier-orbital interactions in dissociation and substitution reactions with metal-specific X-ray spectroscopy, and metal oxidation states with femtosecond pulses for ‘probe-before-destroy’ spectroscopy. These are considered to be of relevance because they may hold the key to understanding and controlling how the reactivity of 3d transition-metal systems can be exploited to convert the initially absorbed photon energy with optimized rate and selectivity into other forms of energy.

**Interlude.** *So why are these four topics suited to evaluate the role of X-ray free-electron lasers and how does this relate to the idea that ‘things are coming together’? To illustrate this I am using an example that I happen to know reasonably well and this concerns the very simple 3d transition-metal complex iron pentacarbonyl, Fe(CO)_*5*_. Ever since Sir James Dewar and Humphrey Owen Jones noted in 1905 that ‘in the laboratory on bright days in February the decomposition was extremely slow, but on the same days in direct sunlight the decomposition was rapid’* [34] *it has been known that iron pentacarbonyl decomposes and releases carbon monoxide when exposed to visible light. This basically marks the beginning of the investigations of the photochemistry of this molecule. It was rationalized in the 1960s that the extreme photosensitivity of iron pentacarbonyl and other metal carbonyls had to be due to the substantial charge-density changes generated by exciting electrons in the strongly covalent metal-carbon monoxide bonds* [35]*. Irrespective of the photochemistry of Fe(CO)_*5*_, molecular-orbital theory was developed in parallel and one of the first molecular-orbital diagrams published by Harry B. Gray was actually the one of iron pentacarbonyl* [36]*. The connections between the photochemistry and molecular-orbital theory of carbonyls was readily recognized as it offered a conceptual way to describe and quantify the charge-density changes upon electronic excitation. Accordingly, one of the first reviews summarizing the photochemistry of metal carbonyls by Mark Wrighton from the 1970s included the concept of molecular-orbital theory* [37]*. Still, understanding the photodissociation of iron pentacarbonyl was complicated by its elusive excited-state dynamics. Early electronic-structure calculations from the 1980s by Chantal Daniel accordingly indicated the importance of accounting for spin barriers resulting from different multiplicities in the electronically excited states of the complex* [38]*. In this respect the photodissociation of Fe(CO)_*5*_ has always been a prototypical example for understanding how the reactivity in organometallic photoreactions and the dynamics of charge and spin densities are correlated. In fact iron pentacarbonyl made its way into modern text books such as into the one by Albright* et al. *entitled* Orbital Interactions in Chemistry [39]*. Various time-resolved methods were then used to probe the short-lived intermediates involved in the photochemistry of iron pentacarbonyl and the paper by Poliakoff and Turner from 2001 summarized the widely accepted state-of-the-art* [40]*. One of the main and maybe one of the most obvious questions, however, remained open, namely, how does the iron-carbonmonoxide bonding actually change in the course of the photochemical reaction? With all the information available from the different subfields of, amongst others, molecular-orbital theory, spin states in excited-state dynamics, and time-resolved probing of reaction intermediates each concentrating on a different aspect of the same problem, the question really is why that question remained open? This is where everything goes back to Dewar. Because this is where one necessarily ends up when going back in time, passing the points back in time where the problem bifurcated into the different subfields or disciplines. Dewar wrote in his paper that ‘the initial action of light on iron carbonyl might be represented by the equation Fe(CO)_*5*_ = Fe(CO)_*4*_ + CO’* [34]*. So what does this equation actually mean? What is behind it? Or how does bonding, how do charge and spin densities actually change as iron pentacarbonyl dissociates? This may be regarded the question unifying all different aspects of the same problem and this is where X-ray spectroscopy and XFELs come in. X-ray spectroscopy probes chemical shifts of core-level binding energies as demonstrated in the work by Kai Siegbahn in the 1970s* [41]*. Because the chemical shift can be thought to depend on charge and spin densities at the probed element and chemical site, X-ray spectroscopy may give access to charge and spin densities. Femtosecond pulses from XFELs render snapshots of short-lived intermediates and may give access to how charge and spin density change in the course of the reaction. Asking the unifying question about how charge and spin densities transiently change in the photochemical reactions of 3d transition-metal systems means ‘going back in time and passing the bifurcations' where subfield developed and the different aspects got separated. Because XFELs can be used to probe different aspects of the same problem, they may give answers to this unifying question and ‘bring back together’ different aspects of the same problem*.

## 2. Structural dynamics with metal K-edge absorption spectroscopy

XAS at the K-edges of 3d transition metals is a widely used tool to characterize the structure of transition-metal complexes and metalloproteins [2]. One often distinguishes the extended X-ray absorption fine structure (EXAFS) due to scattering of the liberated metal 1 s electron by the surrounding neighbours and the X-ray absorption near edge structure (XANES) due to, in a simplified picture, specific excitations of the metal 1 s electron into molecular orbitals [2]. For EXAFS, an extended energy range above the absorption edge is scanned to probe structure (such as number and distances of neighbours) while for XANES the spectral features near the absorption edge are analysed to probe a combination of geometric and electronic structure effects. In addition, the so-called pre-edge features below the edge can be analysed. They are due to quadrupolar transitions between the metal 1 s and the lowest unoccupied metal 3d-derived molecular orbitals and give direct access to the electronic structure. Because current short-pulse X-ray sources offer very limited capabilities for time-resolved EXAFS, I focus here on XANES and for simplicity call it XAS. The suitability of time-resolved K-edge XAS to probe the structural dynamics of transition-metal complexes and metalloproteins has been recognized early on and the pioneering work by Bressler & Chergui [42] and Chen *et al.* [18] and others is well reviewed by Chergui & Collet [2] including work with ps temporal resolution at synchrotron radiation sources and pioneering studies in the fs domain at XFELs.

**Figure 1.**
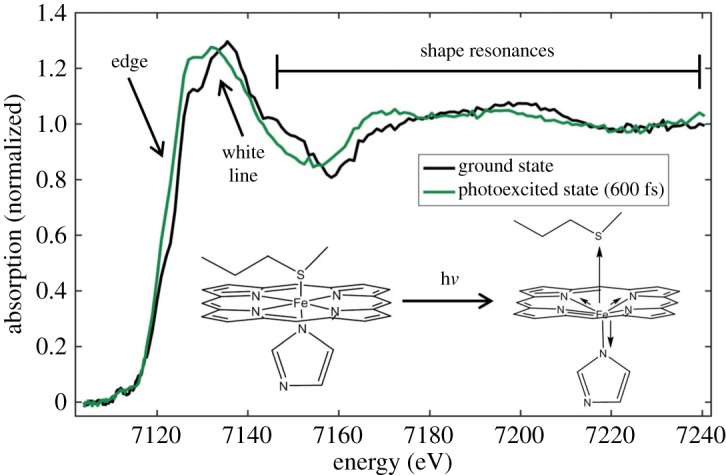
Time-resolved X-ray absorption spectroscopy results of the ferrous (Fe^II^, Fe^2+^) horse heart cytochrome c metalloprotein. The Fe K-edge absorption spectrum of ground state cytochrome c is compared to the spectrum of the photoexcited state measured at a time delay of 600 fs between optical pump and X-ray probe pulses. Important spectral differences between the spectra are indicated for the ‘edge’ energy, the main absorption line (white line) and the so-called shape resonances above the absorption edge. Insets show structural models schematically depicting the structural changes induced by photoexcitation (h*ν*). Reproduced with permission from Mara *et al*. [43] (Copyright © 2017 AAAS). (Online version in colour.)

Here, I use the study by Mara *et al.* from the Solomon group [43] with measurements from the Linac Coherent Light Source (LCLS) XFEL to demonstrate the capabilities of fs time-resolved K-edge XAS at XFELs. One of the main results of this study is depicted in figure 1. The authors studied the cytochrome c metalloprotein that contains in its active site a six-coordinate low-spin heme iron. They investigated how cytochrome c switches between two completely disparate functions: It can act as an electron-delivery system (as an electron-transfer agent in respiration) by adapting different iron oxidation states without changing its spin state and coordination. Alternatively, it can act as a catalytic reactant (as a peroxidase in apoptosis, a form of programmed cell death) by dissociating an amino acid ligand (Met80 with an iron-sulfur bond in the Fe-S(Met80) configuration) where ligand dissociation opens a binding site for the reaction [43,44]. The Fe-S(Met80) ligand dissociation can be initiated by absorption of visible light and it takes place on a time scale of below 1 ps, followed by geminate recombination on a time scale of several ps. Key to elucidating how cytochrome c switches function (by quantifying the energetic cost of the so-called entatic stabilization of the weak Fe-S(Met80) bond in the protein) was to characterize the geometric and electronic structures of the transient intermediate iron species from the ligand dissociated species and back to the reformed ground state. This was achieved by optical pump and X-ray spectroscopy probe measurements enabled following the changes in active iron site conformation in time. The Fe K-edge XAS spectrum in figure 1 of photoexcited cytochrome c 600 fs after optical pumping and when the ligand has dissociated (see structural models in the inset of figure 1) exhibits characteristic changes compared to the ground state spectrum of the non-dissociated species. The edge energy shifts to lower energies and intensity in the edge increases, the white line (main absorption line) shifts to lower energies and the intensity maximum in the so-called shape resonance region shifts to lower energies. Because these changes are so characteristic and by comparison to calculated spectra (not shown) the authors could readily conclude from these changes that the active site in cytochrome c converts from the low-spin six-coordinate ground state to a high-spin five coordinate Fe^II^ species in the photoexcited state. The Fe-S(Met80) bond length is found to increase from 2.3 to 2.9 Å where the latter can be considered broken with concomitant increase of the remaining Fe-N bond lengths. The sensitivity of Fe K-edge XAS to the ligand environment of Fe was essential here to verify the ligand loss upon photoexcitation.

**Figure 2.**
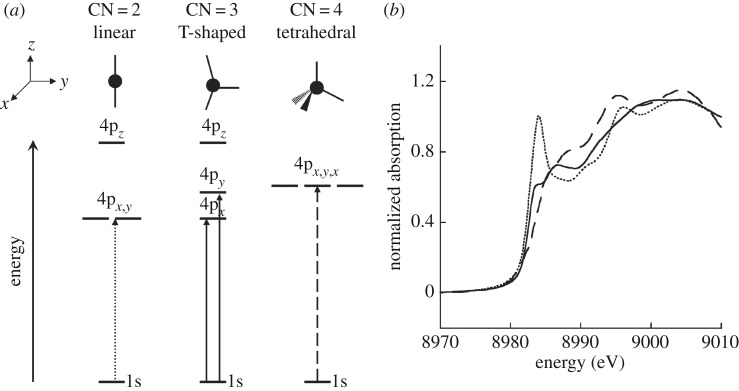
Schematic illustration and measurements for how structure, geometry and coordination are correlated in metal K-edge absorption spectroscopy. (*a*) Ligand field splitting and (*b*) metal K-edge absorption spectra for exemplary Cu^I^ (Cu^1+^) centred model complexes. The Cu K-edge absorption spectra (*b*) correspond to different Cu complexes (*a*) with different coordination numbers (CN) of the Cu centre with CN = 2 (dotted), 3 (solid) and 4 (dashed). The peak at approximately 8985 eV in the spectra is due to Cu 1s → 4p transitions that can be interpreted to probe the energies of the metal 4p-derived molecular orbitals. Changes in energy, intensity and shape of this peak reflect changes in ligand-field splitting as the coordination of the Cu centre changes. The ‘energy’ in (*a*) corresponds to orbital energies and increases from bottom to top as indicated (binding energies thus increase from top to bottom). Reproduced with permission from Solomon *et al*. [45] (Copyright © 2013 Elsevier).

**Figure 3.**
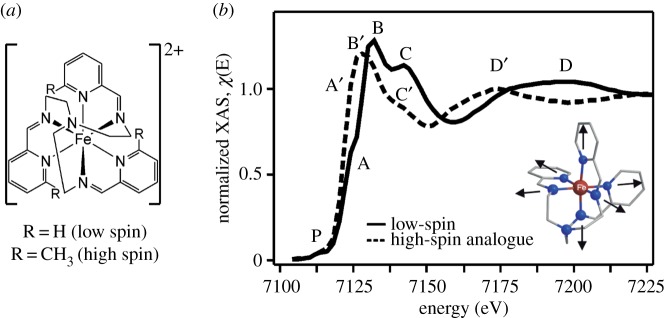
An exemplary case of ferrous Fe^II^ (Fe^2+^)-centred model complexes for how structure (specifically Fe-ligand bond length) and Fe spin state are correlated with Fe K-edge absorption spectra. (*a*) Ligand structure of the cation in two Fe^II^ complexes with a low-spin singlet (t_2_^6^e^0^) configuration for R = H and a high-spin quintet (t_2_^4^e^2^) configuration for R = CH_3_. (*b*) Fe K-edge absorption spectra of the two complexes with indicated regions of characteristic spectral changes. The average Fe-N bond length is larger by 0.23 Å in the high-spin compared to the low-spin complex and a transient Fe-N bond length increase (see inset in *b*) was measured by Khalil *et al*. in this study with time-resolved Fe K-edge absorption spectroscopy (not shown here). Adapted with permission from Khalil *et al*. [46] (Copyright © 2006 American Chemical Society). (Online version in colour.)

The here employed structural sensitivity of metal K-edge XAS has been established in numerous steady-state studies and the changes in the edge, the white line and in the shape resonance region are detailed in figures 2 and 3. The seminal investigation of Cu^I^ complexes by the Solomon group shown in figure 2 [45,47] establishes how changes in metal coordination and ligand-field splitting are reflected in the edge energy and intensity and the cytochrome c spectra in figure 1 can be qualitatively understood based on this. The edge energy and intensity can be thought to be determined by (dipole allowed) transitions between the metal 1 s and the lowest unoccupied metal 4p-derived molecular orbitals (1s→4p transitions, figure 2), where the unoccupied molecular orbitals result from interactions of metal 4p and ligand p orbitals (2p for C, N, O and 3p for S) [45]. Reducing coordination (such as by ligand dissociation) reduces covalent interactions which in turn increases the amount of p-character in the 4p-derived molecular orbitals and the intensity in the edge increases (figure 2). At the same time and approximately within this one-electron molecular-orbital picture and based on qualitative considerations of the involved ligand fields [47], the energy of the 4p-derived molecular orbital decreases thereby reducing the 1s→4p edge energy. Changes in energies and intensities of peaks in the K-edge XAS spectrum were also used by the group of Chergui in a systematic time-resolved K-edge XAS study at a synchrotron radiation source of a Cu-phenantroline complex [48] and by the group of Chen to reveal the ultrafast excited-state dynamics of a Ni porphyrin complex with fs resolution Ni K-edge XAS at an XFEL [49].

Still with the aim to understand the shifts of the white line and shape resonances to lower energies in the cytochrome c spectra in figure 1, it has to be noted that the situation is complicated by that in addition to ligand dissociation, the system is thought to convert from a low-spin to a high-spin Fe^II^ species in the photoexcited state. Interconversion of metal complexes between different spin states was studied in a number of seminal time-resolved Fe K-edge XAS studies of Fe^II^ complexes where optical excitation was used to trigger low-spin to high-spin conversion [2,17,46,50,51]. In addition to a comparison to calculated spectra, the interpretation can be based on the comparison of steady-state Fe K-edge XAS spectra of chemically (instead of photochemically) prepared low-spin and high-spin Fe^II^ complexes where similar spectral changes are observed [46,52,53]. Figure 3 summarizes the exemplary study on this by the Schoenlein group by Khalil *et al.* [46]. It demonstrates how the white line and shape resonance energies shift to lower energies upon conversion from chemically prepared low-spin to high-spin Fe^II^ complexes. Similar to the Cu^I^ case in figure 2, these changes can be readily assigned to an elongation of the Fe-N bonds by approximately 0.2 Å in the high-spin compared to the low-spin complex: An increase in the Fe-N bond lengths with a reduction of covalent interactions and a decrease of 4p-derived molecular orbital energies explains the edge intensity increase (A/A′ in figure 3) and the red shift of the white line (B/B′ in figure 3). With increasing incident photon energy approaching the EXAFS region in the K-edge spectrum the influence of scattering effects on intensity and energy of spectral features (such as feature C/C′ in figure 3) complicates their assignment rendering the extraction of unambiguous information difficult. The shape resonances (D/D′ in figure 3) were proposed to arise from scattering of the metal 1 s electron from surrounding atoms. This is based on the seminal work by Farrel Lytle and co-workers [54] (after whom the ‘Farrel W. Lytle Award’ of the Stanford Synchrotron Radiation Lightsource (SSRL) facility at the SLAC National Accelerator Laboratory in Stanford is named). Following this work, a rule now known as Natoli's rule was proposed [55]. According to this, the energy shift of the shape resonance can be used to estimate changes in distances between the probed metal and its nearest neighbours because the energy of the shape resonance can be thought to approximately depend on the nearest-neighbour distances *R* by 1/*R*^2^. Concerns about limitations of this rule have been raised repeatedly [53,56] and they all relate to the complications arising from the fact that both geometric and electronic structure effects considerably influence energies and intensities of the transitions in the transition-metal K-edge XAS spectra.

There are several ways to probe the electronic structure of 3d transition-metal complexes more directly and one is to analyse the pre-edge features in K-edge XAS spectra located at energies below the edge (they are seen at approx. 7110 eV in figure 1 and labelled P in figure 3). These are due to 1s → 3d transitions [45,57] and they give direct access to the 3d-derived frontier orbitals. Because these transitions are dipole forbidden they are much weaker than the transitions in or above the edge and this has largely hampered their utilization so far in time-resolved K-edge XAS studies of transition-metal complexes and metalloproteins at XFELs (with first indications of the usefulness by Chen's group [49] and in a study by Lemke *et al.* [58] as well as in exemplary studies at synchrotron radiation sources such as the Ti K-edge XAS study of TiO_2_ by the Chergui group [59]). Other ways of probing the electronic structure more directly are Kβ x-ray emission spectroscopy and L-edge spectroscopy discussed in the following sections. Before proceeding to this, I want to briefly present two further studies of structural dynamics of 3d transition-metal complexes with time-resolved K-edge XAS because of their innovative character.

**Figure 4.**
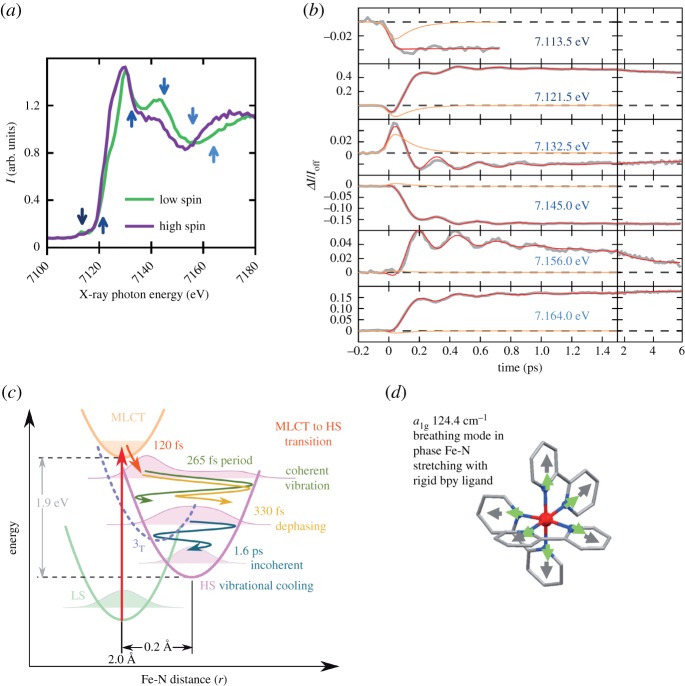
Time-resolved Fe K-edge absorption spectroscopy results on the excited-state dynamics upon spin-state conversion in the low-spin Fe^II^ complex [Fe(bpy)_3_]^2+^. (*a*) Fe K-edge XAS spectra of the low-spin ground state and the high-spin excited state (measured at a time delay between optical pump and X-ray probe pulses of 10 ps). (*b*) Time delay scans (grey lines) measured at the indicated photon energies (relative absorption changes as a function of pump-probe delay time, *ΔI/I*_off_ is the intensity change *ΔI* with respect to the intensity without optical pump *I*_off_, red lines are fitted curves from a global fit to all data, orange curves represent the population of the initially photoexcited MLCT state, see (*c*). (*c*) Schematic energy-potential diagram of the photoreaction of [Fe(bpy)_3_]^2+^ (for details on the dephasing of the wavepacket and the transition to incoherent vibrational cooling see the original publication). (*d*) Structural representation of the normal breathing mode of the molecule (period 265 fs) as detected in the experiment (red: Fe, blue: N, grey: bipyridine ligands, green arrows: motions of N atoms, grey arrows: motions of the bipyridine ligands). Adapted from [58] (licensed under a Creative Commons Attribution 4.0 International License, http://creativecommons.org/licenses/by/4.0/). (Online version in colour.)

The study by Lemke *et al.* [58], summarized in figure 4, reports an Fe K-edge XAS study at the LCLS XFEL of a low-spin to high-spin conversion in an Fe^II^ complex as triggered by optical excitation. The spectral changes (figure 4*a*) that are very similar to the previously discussed changes (figure 3) were measured at an XFEL with a temporal resolution of only 60 fs (FWHM). In contrast to previous time-resolved X-ray spectroscopic studies of this and similar complexes, this high temporal resolution together with an unprecedented signal-to-noise ratio made visible damped oscillations in the absorption intensities (figure 4*b*) between 200 fs and several ps after optical excitation. These oscillations appear for energies at or above the edge where the K-edge spectrum exhibits structural sensitivity. Note that the pre-edge intensities, sensitive to electronic structure, do not exhibit such oscillations here in this case (figure 4*b*, top trace) and future studies will elucidate the benefit of investigating these pre-edge features in time-resolved K-edge XAS [60]. Lemke *et al.* found that the Fe^II^ system, after having been excited from the low-spin ground state to a low-spin metal to ligand charge transfer (MLCT) manifold of states, transits to a high-spin state within about 120 fs (figure 4*c*). It is the coherent vibrations of the complex in this high-spin state (figure 4*c*) that create the observed intensity oscillations thereby complementing earlier reports based on all-optical methods [61,62]. With a detailed analysis of the structural sensitivity of K-edge XAS at the various energies probed in the experiment the oscillations were assigned to a breathing mode of the complex (oscillation period 265 fs) with in-phase stretching of the Fe-N bonds with rigid bipyridine (bpy) ligands (figure 4*d*). The origin of the reported spectral changes is thus the same as for the previously discussed cases (elongation of the Fe-ligand bond length) but the detection of the coherent vibrations cannot be overrated as it allows identifying one of the key reaction coordinates in the excited-state dynamics of this complex (Fe-N distance). Only with this information can the abscissa in the energy-potential diagram in figure 4*c* with units in Å in fact meaningfully be assigned and the excited-state dynamics effectively be interpreted. The reported sensitivity thereby gives access to essential relaxation phenomena in excited-state dynamics of 3d transition-metal complexes such as vibrational cooling and the dephasing of nuclear wavepackets that are indispensable for understanding the excited-state dynamics of transition-metal complexes.

**Figure 5.**
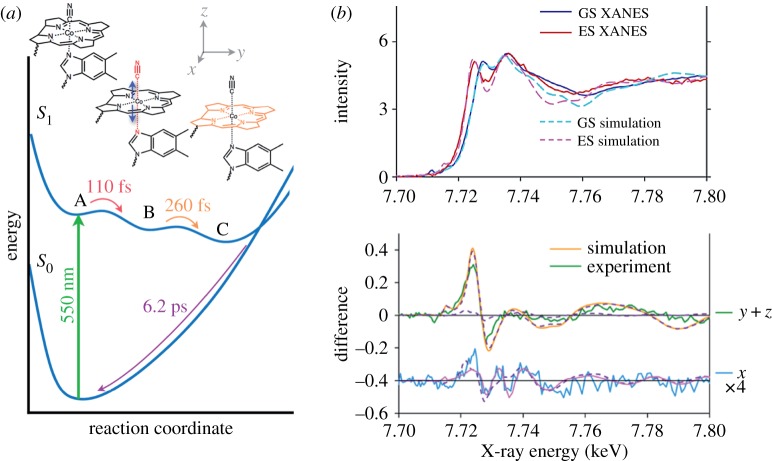
Time- and polarization-resolved X-ray absorption spectroscopy results of vitamin B_12_ cyanocobolamin where measured for different time delays between optical pump and X-ray probe pulses and for varied relative (linear) polarizations of pump and probe pulses. (*a*) Schematic depiction of the energy potential landscape of vitamin B_12_ cyanocobolamin with structural models illustrating the coordination changes around the Co centre during the photoreaction. Electronic excitation to the lowest excited *S*_1_ state at 550 nm (structure A) corresponds to *π* → π* transitions in the corrin ring (N-anchored ring surrounding the Co centre) with subsequent elongation of the axial Co-CN bonds after 110 fs (to structure B), relaxation of the corrin ring with increasing distances of the equatorial Co-N (N in the corrin ring) bonds after 260 fs (to structure C) and internal conversion to the ground state after 6.2 ps. (*b*) (top) Comparison of calculated and measured Co K-edge absorption spectra (denoted XANES here for X-ray absorption near-edge structure) of ground state (GS) cyanocobolamin and excited-state (ES) cyanocobolamin (structure C as measured at a time delay between optical pump and X-ray probe pulses of 1.5 ps). (*b*) (bottom) Polarization-resolved difference between ground- and excited state spectra with separate *y* + *z* and *x* components (coordinate system see (*a*), green: measured *y* + *z*, yellow: simulated *y* + *z* with separate *y* and *z*, both dashed, *z* being larger than *y*, blue: measured *x*, pink: simulated *x* with simulated *y*, dashed, overlaid). Adapted with permission from [63] (Copyright © 2017 American Chemical Society). (Online version in colour.)

Finally, and as a last example for studies of structural dynamics of metal complexes with K-edge XAS, one of the main results of a study from the Sension group based on measurements at the LCLS XFEL on the excited-state dynamics of the Co-centered vitamin B_12_ cyanocobolamin by Miller *et al.* is shown in figure 5 [63]. As one of the first groups, if not as the first group ever, the authors resolve or control, in addition to time, in an optical pump and X-ray spectroscopy probe scheme, the relative polarizations of pump and probe beams. The sample is isotropic (in the sense that it is not actively pre-aligned or pre-oriented) but the electronic transition at the chosen pump wavelength of 550 nm is anisotropic in that the electric transition dipole lies in the plane of the corrin ring surrounding the Co centre (figure 5*a*). Because optical excitation is stronger for those molecules in the beam where the transition dipole is parallel to the optical laser polarization, a sub-ensemble of molecules is selected by excitation (it is noteworthy that rotational diffusion that could counteract this pre-selection of similarly aligned/oriented molecules does not influence the data because it takes much longer than the lifetime of the photoexcited state of several ps, see [63]). Similar arguments apply for the X-ray probing: X-ray excitations at the Co K-edge can be thought of as excitations of Co 1 s electrons to continuum states with p symmetry. The corresponding liberated electron thus propagates as a p wave oriented along the polarization axis of the probing X-ray beam. These considerations explain the observed dichroism in the pump-probe signals. The polarization sensitivity enabled the authors to dissect the measured spectral changes into different spatial contributions (figure 5*b*) and this, in turn, allowed assigning measured time constants to specific structural changes or reaction coordinates. In the extracted sequential mechanism for the excited-state dynamics of cyanocobolamin (figure 5*a*), the polarization-resolved spectra in figure 5*b* at a time delay of 1.5 ps after optical excitation characterize the photoexcited structure C: The dominant spectral difference of the *z* component of the Co K-edge XAS spectrum directly demonstrates elongation of the axial Co-ligand bonds in this species (the *z*-direction is out of plane of the corrin ring, see coordinate system in figure 5*a*).

## 3. Spin states in excited-state dynamics with metal 3p-3d exchange interactions

Because it is so hard to unambiguously distinguish between geometric and electronic structure effects on energies and intensities in transition-metal K-edge XAS spectra, other ways for probing the electronic structure of the metal are needed. This section focuses on ways to probe the spin state (metal spin multiplicity) in photochemical reactions of 3d transition-metal complexes and metalloproteins. The sensitivity of the discussed methods (Kβ XES and 3p photoelectron spectroscopy), as different as they are, originates for both from the intra-atomic exchange Coulomb interactions [64], often also denoted simply ‘exchange interactions', between the metal 3p and 3d electrons. It is one intention of this section to elucidate the commonalities between Kβ XES and 3p photoelectron spectroscopy.

The importance of detecting the metal spin state in the excited-state dynamics of transition-metal photochemistry is well illustrated for optically induced spin conversions (ultrafast spin crossover dynamics) of metal complexes in the review by Zhang & Gaffney [65]. A famous and particularly nice example for the detection of spin states of transient intermediates with time-resolved metal Kβ XES is the study by the Gaffney group by Zhang *et al.* [66] on the spin crossover dynamics of [Fe(bpy)_3_]^2+^ (the same complex as studied by Lemke *et al.* and discussed in the preceding section). This study is based on or complements numerous other studies by other authors on the same topic of ultrafast spin crossover dynamics using other complexes (see references in [65] and [2]). The study by Zhang *et al.* also built the basis for subsequent studies focusing on controlling or manipulating the excited-state dynamics of these systems [67,68].

**Figure 6.**
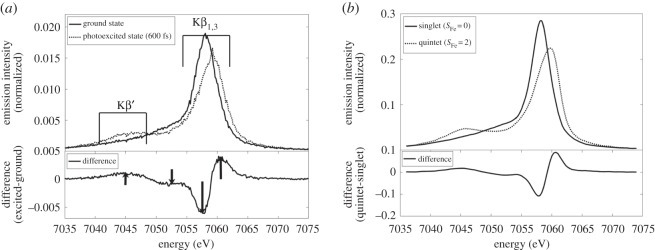
Time-resolved Kβ X-ray emission spectroscopy results of the ferrous (Fe^II^, Fe^2+^) horse heart cytochrome c metalloprotein. (*a*) Fe Kβ emission spectra of the ground state and photoexcited cytochrome c measured at a time delay of 600 fs between optical pump and X-ray probe pulses (the characteristic spectral regions of a 3d transition-metal Kβ emission spectrum are indicated, spectra normalized to the same integrated intensities, difference of the spectra in the lower panel). (*b*) Fe Kβ emission spectra of Fe^II^ reference complexes in low and high spin states with singlet (total spin S at the Fe centre equals 0 with zero unpaired 3d electrons) and quintet (total spin S at the Fe centre equals 2 with four unpaired 3d electrons, spectra normalized to the same integrated intensities, difference of the spectra in the lower panel). Reproduced with permission from Mara *et al*. [43] (Copyright © 2017 AAAS).

Here with figure 6 we come back to the study of Mara *et al.* by the Solomon group on the cytochrome c metalloprotein because this is a particularly nice example of how time-resolved Kβ XES was used to provide the unambiguous information on the spin state of the photoexcited state of the system; information missing or less obvious from K-edge XAS. The spectral differences between the Fe Kβ XES spectra of the ground state and photoexcited cytochrome c in figure 6*a* are compared to the differences between the steady-state Fe Kβ XES spectra of two reference Fe^II^ complexes (figure 6*b*) where one is in a low-spin state and one is in a high-spin state. The close similarity of the characteristic changes throughout the spectrum (see the difference spectra in figure 6*a,b*) unambiguously shows that the photoexcited state of cytochrome c (at the measured delay time of 600 fs after optical excitation) adopts a high-spin state with quintet multiplicity of the Fe centre, which corresponds to a total 3d spin S of 2 and results from four unpaired Fe 3d electrons. This information proved essential for elucidating how cytochrome c switches function and nicely complements the previously discussed verification of ligand-loss in the photoexcited state of cytochrome c by time-resolved Fe K-edge XAS.

**Figure 7.**
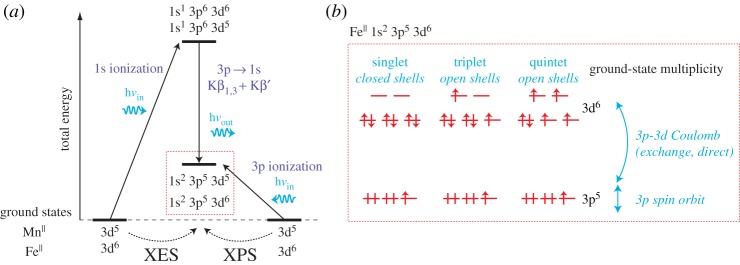
Conceptual depiction of Kβ X-ray emission spectroscopy (XES) and 3p X-ray photoelectron spectroscopy (XPS) probing the same 3p core-hole final states and their sensitivity to the effective number of unpaired 3d electrons. (*a*) Many-electron total-energy diagram (the total energy denotes the energy in the system with respect to the ground state, in contrast to the one-electron orbital-energy diagram in figure 2 and in (*b*)) for Kβ XES and 3p XPS for Fe^II^ 3d^6^ and Mn^II^ 3d^5^ initial ground states. (*b*) One-electron orbital-energy diagram (increasing orbital energy from bottom to top, binding energies increase from top to bottom) of the Fe^II^ 1s^2^ 3p^5^ 3d^6^ final state of (*a*) with an arbitrary valence electronic structure of 3d orbitals for singlet (zero unpaired 3d electrons), triplet (two unpaired 3d electrons) and quintet (four unpaired 3d electrons) multiplicities of Fe^II^ in the initial ground state. The 3p spin-orbit interactions and the 3p-3d Coulomb interactions are indicated (exchange Coulomb interactions for open 3d shells with unpaired 3d electrons, direct Coulomb interactions for 3d shells other than half filled with orbital angular momenta). (Online version in colour.)

Kβ XES is one of several X-ray emission spectroscopic methods and it was well reviewed by Glatzel & Bergmann [69]. As illustrated with figure 7*a*, Kβ X-ray emission of 3d transition-metals results from fluorescence decay of the metal 1 s core-hole states (created by 1 s ionization with the incident X-ray radiation) where a metal 3p electrons fill the 1 s vacancy (3p → 1 s transitions). The resulting final state is a 3p core-hole state (with a 3p vacancy) and atomic multiplet splittings are determined by 3p-3d Coulomb interactions. As indicated in figure 7*b*, for an Fe^II^ initial ground state these intra-atomic (electrostatic) Coulomb interactions are exchange interactions when the 3d shell is open with unpaired 3d spins (as illustrated). Via these exchange Coulomb interactions Kβ XES thus probes the spin state of the metal because the shape of the spectrum critically depends on the number of unpaired metal 3d electrons (which obviously determines the metal spin state).

**Figure 8.**
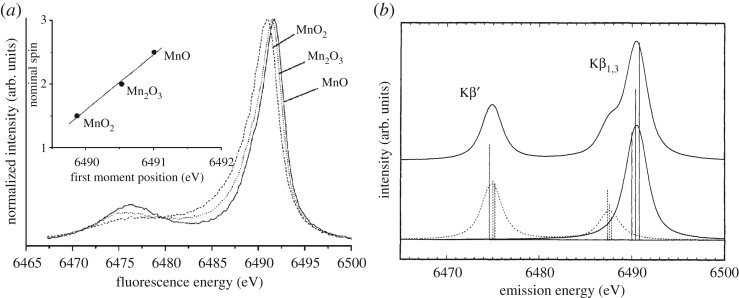
Mn Kβ X-ray emission spectroscopy (XES) as a measure of the number of unpaired Mn 3d electrons. (*a*) Kβ emission spectra of selected Mn oxides with (in the respective ground states) nominal Mn oxidation states of Mn^II^ (MnO), Mn^III^ (Mn_2_O_3_) and Mn^IV^ (MnO_2_) and effective numbers of unpaired Mn 3d electrons of 5 (MnO, total 3d spin 5/2), 4 (Mn_2_O_3_, total 3d spin 2) and 3 (MnO_2_, total 3d spin 3/2). The peak at 6490 eV is denoted Kβ_1,3_ and the peak at 6475 eV is denoted Kβ’ (the inset shows the first moment positions of the main Kβ_1,3_ peak with a linear fit). (*b*) Atomic multiplet calculation of the Mn^II^ Kβ emission spectrum (Mn^II^ 1s^1^ 3p^6^ 3d^5 ^→ Mn^II^ 1s^2^ 3p^5^ 3d^5^ transitions) with, at the top, the total spectrum (solid line) and at the bottom the different components contributing to the spectrum (different groups of final states distinguished according to their total spin) with high-spin (solid line and solid vertical sticks) and low-spin components (dotted line and dotted vertical sticks). In the Mn^II^ 1s^2^ 3p^5^ 3d^5^ final states and within the Russel–Saunders (or LS) coupling scheme of angular momenta (in this scheme the LS terms ^2S+1^L_J_ denote the total spin S and the total angular momentum L with the total angular momentum J of the respective states) the high-spin Kβ_1,3_ components at 6490 eV correspond to states with ^7^P (total spin of S = 3 with spins of the 3p^5^ and 3d^5^ shells oriented parallel) and the low-spin Kβ’ components at 6475 eV correspond to states with ^5^P (total spin of S = 2 with spins of the 3p^5^ and 3d^5^ shells oriented antiparallel, the peak at 6488 eV corresponds to low-spin final states with parallel 3p and 3d spins but where one 3d spin has flipped such that, in contrast to the main lines where all five 3d spins are up, only four 3d spins are up and one is down, spin-orbit interaction in the 3p shell split the LS states ^7^P and ^5^P according to J into three states ^7^P_4,3,2_ and ^5^P_3,2,1_ and these are the three sticks in each of the calculated lines). The vertical sticks were broadened by a 1 eV Lorentzian profile reflecting lifetime broadening of the 1 s core-hole states combined with an 0.5 eV FWHM Gaussian broadening reflecting the experimental bandwidth. (*a*) Reproduced with permission from [69] (Copyright © 2005 Elsevier). (*b*) Adapted with permission from [70] (Copyright © 1994 American Chemical Society).

This is best detailed with the Mn Kβ XES spectra from the seminal studies by Glatzel & Bergmann [69] and by the Cramer group by Peng *et al.* [70] in figure 8. The Mn Kβ XES spectra of Mn^II^, Mn^III^ and Mn^IV^ species in figure 8*a* establish the characteristic spectral changes (comparable to the ones in the Fe Kβ XES spectra in figure 6) and directly correlate (linear scale, see inset in figure 8*a*) with the changing number of unpaired Mn 3d electrons in the compounds from five (Mn^II^ 3d^5^), to four (Mn^III^ 3d^4^) and three (Mn^IV^ 3d^3^). The calculated spectrum in figure 8*b* for the case of Mn^II^ 3d^5^ (and the other cases can be equivalently described) now clearly shows that the shape of the Kβ XES spectrum is mainly determined by local intra-atomic multiplet effects in the final 3p core-hole state as probed by Kβ XES (this is an excited state with an electron missing in the 3p shell as evidenced by the electron configuration of this state 1s^2^ 3p^5^ 3d^5^ and the general nomenclature would be 1s^2^ 3p^5^ 3d^n^ with *n* = 3, 4, 5 for the examples discussed here). In a simplified but valid picture, the spectral changes in the Kβ XES spectrum upon varying the number of unpaired Mn 3d electrons directly correspond to a shift of the main Kβ_1,3_ line which in turn results from a variation of the energetic splitting of the Kβ_1,3_ and the weaker Kβ’ line in the spectrum. The splitting of these two lines of more than 10 eV (figure 8) is determined by the 3p-3d exchange Coulomb interactions because this interaction separates states with parallel and antiparallel 3p and 3d spins (note that the spin of the 3p^5^ shell corresponds to the spin ½ of the 3p hole or vacancy and this then needs to be coupled to the total spin of the 3d electrons, figure 8*b*, see [64] for an introduction to and details of the appropriate Russel–Saunders or LS-coupling scheme). For Mn^II^ (figure 8*b*) the Kβ_1,3_ line corresponds to septet final states with parallel orientation of 3p and 3d spins and is therefore denoted high-spin component. The Kβ’ line corresponds to quintet final states with antiparallel orientation of 3p and 3d spins and is denoted low-spin component (figure 8*b*). The spin-orbit interaction in the 3p shell (figure 7*b*) further splits these lines into three multiplet components each which are not resolved in the XES spectra because the splitting is in the order of or below 1 eV. The 3p-3d exchange Coulomb interactions vary in strength with the number of unpaired 3d electrons: With less unpaired 3d electrons they decrease and this reduces the Kβ_1,3_-Kβ’ splitting (figure 8*a* for Mn^II^, Mn^III^ and Mn^IV^) until, for singlet states with no unpaired 3d electrons, the Kβ’ and Kβ_1,3_ lines start to merge (figure 6 for Fe^II^). This explains the sensitivity of Kβ XES to the spin state of the probed metal.

**Figure 9.**
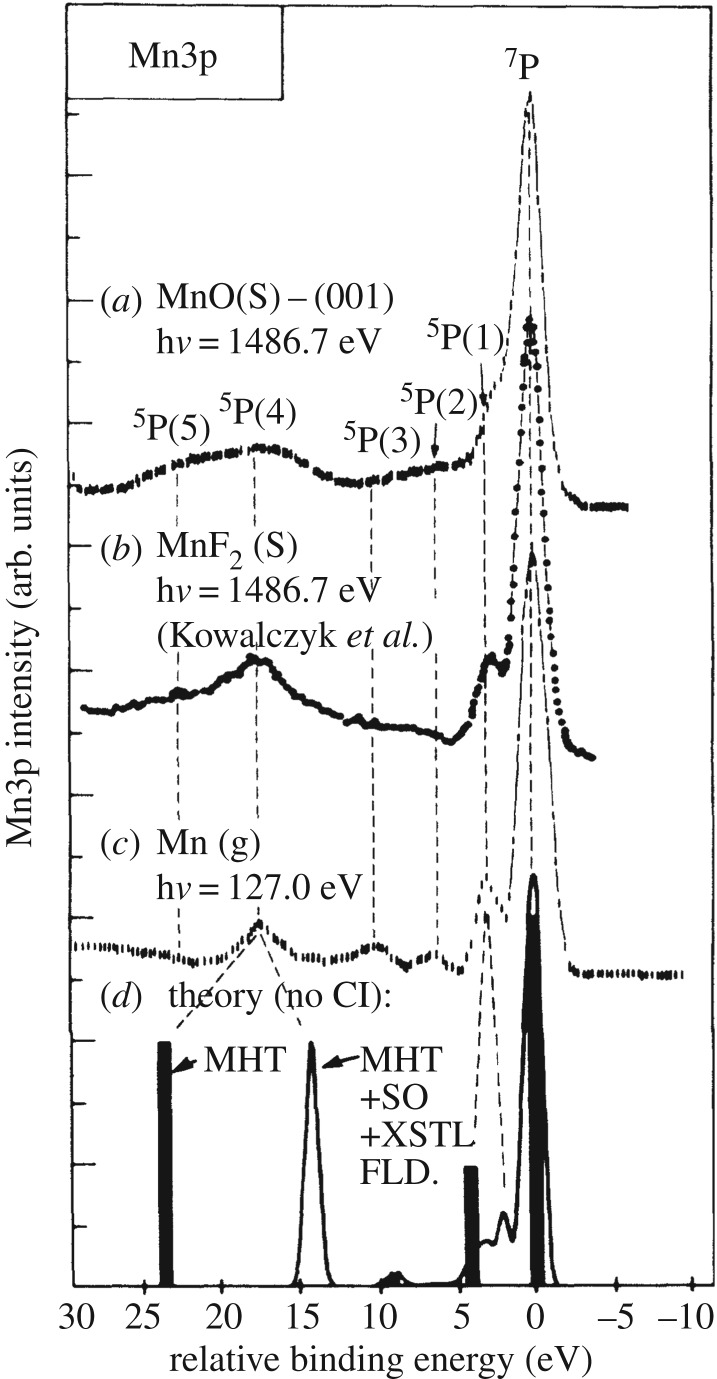
Measured and calculated 3p photoelectron spectra (XPS) of MnO, MnF_2_ and gas-phase atomic Mn (two types of calculations for Mn^2+^ shown as vertical sticks and convoluted spectrum). Note that the binding-energy scale here is inverted with respect to the one plotted in figure 10. Reproduced (figure) with permission from [72] (Copyright © 1988 American Physical Society).

It is intriguing to realize that the final states reached in Kβ XES and the final states of 3p XPS, where the 3p electron is directly ejected into the continuum [71], are identical. This can be easily seen with the state diagram in figure 7*a* and was pointed out before by Glatzel & Bergmann [69]. Furthermore, the Mn 3p XPS spectra from the seminal studies by Fadley and co-workers in figure 9 are ideally suited to support this point [72–74]. First though, note in figure 9 the close similarity of the Mn 3p XPS spectra of MnO, MnF_2_ and gas-phase Mn (in particular the energetic splittings are identical). This clearly demonstrates that the spectrum is dominated by local intra-atomic effects largely independent of the chemical environment. This supports the notion of using 3p XPS or, somewhat equivalently, Kβ XES for probing the electronic structure independent of structural effects. Second, the main peaks in the Mn 3p XPS spectra (^7^P, ^5^P(1) and ^5^P(4) in figure 9) can be readily identified with the peaks in the Mn Kβ XES spectra in figure 8 (^7^P, high-energy ^5^P and low-energy ^5^P in figure 8*b*) as they exhibit the same energetic splitting and are assigned to the same multiplet final states (the additional peaks in the XPS spectra are visible because the spectrum is less broadened than the XES spectrum by lifetime broadening and these peaks result from additional atomic multiplet effects not considered in the calculation in figure 8*b* and not further discussed here [71]. Suppression of the low-spin ^5^P component by term-dependent lifetime broadening due to super-Coster-Kronig decays further complicates the spectra [75] and is not accounted for in the discussion here (see [69] and [71] for more details).

**Figure 10.**
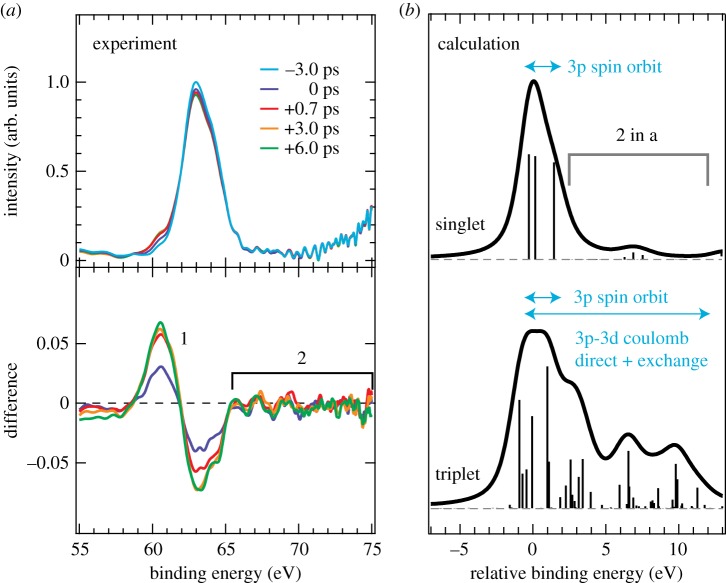
Time-resolved Fe 3p X-ray photoelectron spectroscopy (XPS) of Fe(CO)_5_ and photoproducts and sensitivity to the Fe spin state in the initial ground state. (*a*) Measured Fe 3p photoelectron spectra at given time delays after excitation at 266 nm (incident photon energy 123 eV, intensity of the 3 ps spectrum normalized to one at maximum, differences plotted in the lower panel). (*b*) Calculated Fe 3p photoelectron spectra (crystal-field multiplet, CFM, model) for singlet and hypothetical triplet states of Fe(CO)_5_ and photoproducts (sticks are calculated binding energies and transition intensities of the final ionic 3p core-hole states, spectra aligned to 0 eV for an easier comparison). Note that the binding-energy scale here is inverted with respect to the one plotted in figure 9. Adapted with permission from [76] (Copyright © 2017 AIP Publishing). (Online version in colour.)

With the same final states and with the same origin making both Kβ XES and 3p XPS sensitive to the spin state of the probed metal, applying time-resolved metal 3p XPS to study the spin states of transient intermediates in the photochemical reactions of 3d transition-metal complexes is readily motivated. One such example is our own application of time-resolved Fe 3p XPS measured at the FLASH FEL to study the dissociation of Fe(CO)_5_ in the gas phase [76] summarized in figure 10. Optical excitation of Fe(CO)_5_ at 266 nm was used to trigger dissociation of multiple CO ligands of Fe(CO)_5_ in a sequential process with dominant contributions of Fe(CO)_4_ at time delays of 0.7 ps and Fe(CO)_3_ at 6 ps [77]. A fundamental question in the photochemistry of this and related carbonyl complexes is whether the photoproducts occur in low- or high-spin states during their excited-state dynamics. The Fe 3p XPS spectra measured for various time delays after optical excitation in figure 10*a* clearly exhibit transient changes as a function of time (these are analysed in detail and interpreted in terms of transient chemical shifts in [77]). Importantly for here, however, intensity does not occur for any of the measured delays on the high-binding energy side of the main line in the Fe 3p XPS spectrum (region 2 in figure 10*a*). Intensity would have to arise there if any of the photoproducts occurred in a high-spin state as demonstrated with the calculated Fe 3p photoelectron spectra for low- and high-spin states in figure 10*b*. The 3p spin-orbit interactions splitting the main Fe 3p line for singlet states and the additional 3p-3d Coulomb interactions for open-shell high-spin states leading to further multiplet splitting and intensities at higher binding energies compared to the main line are indicated in figure 10*b*. The absence of intensities characteristic for open-shell high-spin systems was used here to ascertain that Fe(CO)_4_ and Fe(CO)_3_ are in singlet states (as the ground state of Fe(CO)_5_ is) and this is important to understand the role of hypothetical spin-barriers in the excited-state reaction dynamics of this and other carbonyls as triplet states are les reactive than singlets.

## 4. Frontier-orbital interactions in dissociation and substitution reactions with metal-specific X-ray spectroscopy

**Figure 11.**
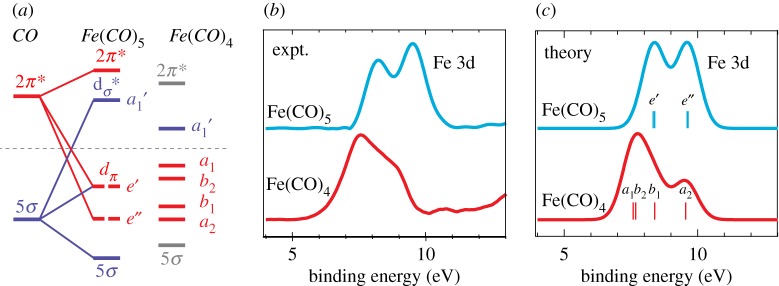
Time-resolved valence photoelectron spectroscopy probing changes in frontier-orbital interactions upon ligand dissociation (CO) from a metal complex (Fe(CO)_5_). (*a*) Molecular-orbital diagram with π interactions in red, *σ* interactions in blue, orbital symmetries *e*’, *e*’’ for the geometry of Fe(CO)_5_, and *a*_1_, *b*_2_, *b*_1_, *a*_2_ for the geometry of Fe(CO)_4_ (orbital energies increase from bottom to top). (*b*) Measured and (*c*) calculated valence photoelectron spectra of Fe(CO)_5_ and Fe(CO)_4_ (the photoproduct Fe(CO)_4_ spectrum was measured with optical pump and X-ray probe time-resolved photoelectron spectroscopy at a pump-probe delay time of 0.7 ps when Fe(CO)_4_is the dominant species). Adapted with permission from [77] (Copyright © 2018 AIP Publishing). (Online version in colour.)

Given that X-ray spectroscopy is sensitive to local charge and spin densities, the question occurs whether time-resolved X-ray spectroscopy can be used to, beyond detecting the spin state of the metal, characterize changes in metal-ligand bonding in photochemical reactions of 3d transition-metal complexes. To approach the answer to this question I stick to the dissociation reaction of Fe(CO)_5_ in the gas phase for a moment and discuss our own study of this system with time-resolved valence photoelectron spectroscopy at the FLASH FEL by Leitner *et al.* as summarized in figure 11 (an extension of the previously discussed study focusing on the Fe spin state) [77]. The molecular-orbital diagram in figure 11*a* details how occupied and unoccupied valence orbital energies are thought to change upon dissociation of a ligand from Fe(CO)_5_ to form Fe(CO)_4_. This diagram is based on the seminal work by Harry Gray [36] and Roald Hoffmann [78,79]. It suggests that the energies of the occupied orbitals increase upon dissociation which is intuitively accessible within a simple picture where reducing covalent interactions upon dissociation increases the energies of occupied orbitals. This readily motivates using valence photoelectron spectroscopy to directly probe these occupied molecular orbitals. Within an approximative Koopman picture where increasing orbital energy corresponds to decreasing binding energy, the measured changes in the corresponding valence photoelectron peaks in figure 11*b* with decreasing binding energy directly reflect the expected changes in metal-ligand bonding upon ligand dissociation. It may be noteworthy that one of the occupied Fe 3d-derived metal-centred *d*_π_ molecular orbitals (e″ in Fe(CO)_5_ geometry, *a*_2_ in Fe(CO)_4_ geometry) is not involved in Fe–CO bonding and therefore its energy does not change. The approximate correspondence of energy shifts in the molecular-orbital diagram and the measured photoelectron spectrum is directly confirmed by the *ab initio* calculations of final ionic state energies by Michael Odelius in figure 11*c*. This investigation demonstrates how the spectral changes in valence photoelectron spectra can be linked to changes in the frontier-orbital interactions upon deligation and it lays the foundation for the following discussion.

**Figure 12.**
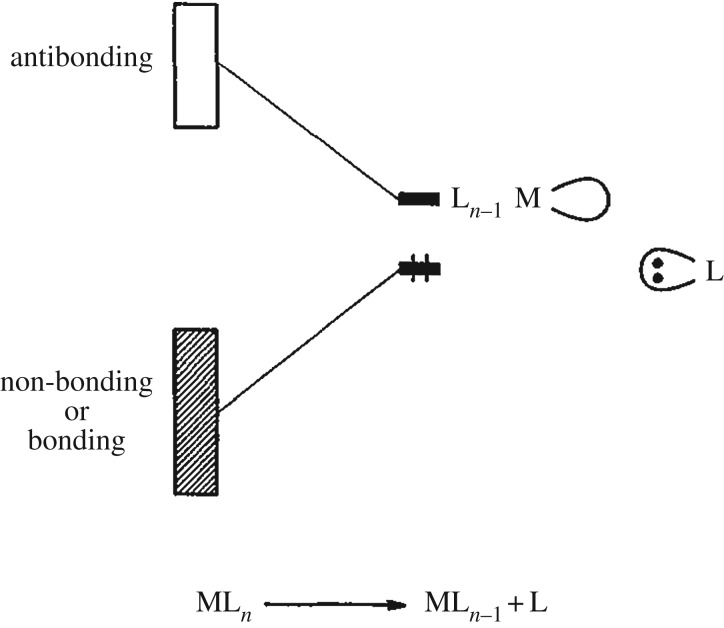
Conceptual depiction of valence electronic-structure changes upon ligand removal from a metal complex. Removing a ligand (L) conceptually corresponds to taking away an electron pair (lobe with two dots on L) from the complex ML*_n_* and creates ML*_n_*_−1_ with a localized hole on the metal (empty lobe on M) and, compared to ML*_n_*, with increases in HOMO (highest occupied molecular orbital) and decreases in LUMO (lowest unoccupied molecular orbital) energies in the dissociated ML*_n_*_−1_ complex. Reproduced with permission from [78] (Copyright © 1982 John Wiley and Sons).

**Figure 13.**
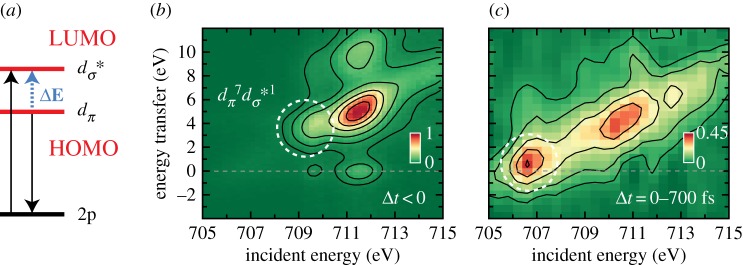
Probing HOMO-LUMO frontier-orbital interactions upon ligand dissociation (CO) from a metal complex (Fe(CO)_5_) with time-resolved metal-specific resonant inelastic X-ray scattering (RIXS) as the X-ray analogue of resonance Raman scattering. (*a*) One-electron orbital-energy diagram of the HOMO (*d*_π_) and LUMO (antibonding d_σ_*) frontier orbitals in Fe(CO)_5_ including the Fe 2p core orbital. Within the approximations of this picture and of the numerous possible transitions at the Fe L-edge (the incident photon energy was scanned across the Fe L_3_-edge), the transitions involving HOMO and LUMO orbitals are discussed here and correspond to Fe 2p→LUMO(d_σ_*) and HOMO(*d*_π_) →2p transitions (arrows for these transitions are indicated and correspond to the incident and scattered photon energies, respectively) thereby probing the HOMO-LUMO energy difference: *Δ*E is the measured difference between incident and scattered photon energies, denoted ‘energy transfer’ here, and it can be approximately identified with the HOMO-LUMO energy difference. With the initial ground state electron configuration *d*_π_^8^d_σ_*^0^ the resulting ligand-field excited final states have *d*_π_^7^d_σ_*^1^ and effectively this corresponds to probing the *d*_π_→*d_σ_* one-electron transitions. (*b*,*c*) Measured Fe L-edge RIXS intensities encoded in colour versus energy transfer and incident photon energy (only the Fe L_3_ edge is shown) of (*b*) ground state Fe(CO)_5_ and (*c*) photoproducts (among others Fe(CO)_4_) as measured with time-resolved RIXS at time delays of 0–700 fs. The final states in the RIXS maps with *d*_π_^7^d_σ_*^1^ configuration are indicated with dashed circles and changes of the energies of intensities in this circle reflect changes of the frontier HOMO (*d*_π_) and LUMO (d_σ_*) orbital energies. Adapted from [80]. (Online version in colour.)

The molecular-orbital diagram in figure 11*a* can be generalized and this was done by Roald Hoffmann with his illustration of the valence electronic structure changes upon deligation in metal complexes in figure 12. The reactivity of unsaturated metal complexes with a missing ligand ML*_n_*_−1_ such as Fe(CO)_4_) can be explained by the electron deficiency at metal (Fe) resulting from the ligand (CO) dissociation. Hoffmann's illustration underlines the importance of the unoccupied (LUMO) orbital for understanding the reactivity of the system as this can be interpreted as a ‘localized hole on the metal’ [78] representing an electronic-structure view of a binding site for reactions. So the question is how to probe these frontier-orbital HOMO-LUMO interactions and the reactive binding site in particular. X-ray photoelectron and emission spectroscopies are ‘blind’ to unoccupied orbitals. XAS can be used to probe unoccupied orbitals but is insensitive to the occupied part of the valence electronic structure (besides being sensitive in addition to geometric structure changes in the case of K-edge XAS). This is where RIXS, combining the sensitivity to occupied and unoccupied orbitals, comes in [10]. Figure 13 summarizes some of the important aspects of our own application of time-resolved RIXS at the Fe L-edge with measurements at the LCLS XFEL to probe the frontier-orbital interactions in the dissociation reaction of Fe(CO)_5_ in solution [80]. For this case (figure 13*a*), RIXS involves the absorption of an incident X-ray photon with a corresponding 2p → d_σ_* one-electron transition from the Fe 2p core orbital to the Fe-centred 3d-derived d_σ_* LUMO orbital. For the transitions I focus on here, inelastic scattering occurs to those final states where an electron from the HOMO (*d*_π_) has filled the Fe 2p vacancy (*d*_π _→ 2p). The resulting energy transfer *ΔE* (difference of incident and scattered photon energy, figure 13*a*) corresponds to, within the approximations of the employed one-electron picture, the HOMO-LUMO energy difference because it effectively probes the HOMO(*d_π_*) → LUMO(d_σ_*) transitions. This establishes the metal-specific probe of the frontier-orbital interactions where the metal specificity is given by the specificity to the Fe L-edge due to the localized nature of the Fe 2p core orbital. In a many-electron total-energy treatment of the process, the RIXS final states can be assigned to ligand-field or charge-transfer valence excited states and numerous examples for steady-state RIXS at the L- and K-edges of 3d transition-metal complexes and metalloproteins are discussed in [10]. In this picture, the transitions discussed here correspond to scattering to ligand-field final states involving exclusively nominally Fe-centred orbitals. These can be approximately related to the discussed orbital excitations. The measured time-resolved RIXS data for Fe(CO)_5_ and photoproducts are depicted in figure 13*b,c*, respectively. The encircled signals can be assigned to the discussed HOMO(*d*_π_) → LUMO(d_σ_*) transitions in Fe(CO)_5_ (figure 13*b*) and Fe(CO)_4_ (figure 13*c*) and the observed decrease in energy transfer from around 3–4 eV in Fe(CO)_5_ to around 0–1 eV in Fe(CO)_4_ can be interpreted as a direct reflection of the decrease in HOMO-LUMO separation as conceptually predicted by Hoffmann (figure 12). The decrease of the incident photon energy for the 2p → LUMO(d_σ_*) transitions from 709 eV in Fe(CO)_5_ to 706–707 eV in Fe(CO)_4_ can as well be understood in the simplified one-electron orbital picture as these transition energies can be expected to decrease concomitantly with the decreasing LUMO(d_σ_*) orbital energy. This demonstrates that time-resolved RIXS effectively enables testing fundamental concepts for how metal-ligand bonding changes during photochemical reactions. The photochemistry of Fe(CO)_5_ in solution is much more complex than what is discussed here and it is complicated in particular by excited-state dynamics of the system with changes of the Fe spin state and possible ligations through geminate recombination with CO or ligation of solvent molecules to the reactive Fe(CO)_4_ species. Detailed analysis of the time-dependent RIXS data led us to propose a reaction mechanism for the excited-state dynamics of the system including some conclusions on how the LUMO (d_σ_*) orbital determines the reactivity of the transient intermediate Fe(CO)_4_ [80,81]. In another case, the approach of time-resolved RIXS was extended to studying the charge-transfer dynamics in a Fe-centred complex [82].

One certainly cannot generalize from so few cases and so far there are two cases only where time-resolved metal L-edge RIXS was used to characterize changes in metal-ligand bonding in photochemical reactions. I still believe that this is a somewhat general concept because extended classes of interesting systems are easily imaginable where this approach could be similarly applicable. One will see whether it proves useful in future investigations of ligand dissociation and substitution reactions as well as charge-transfer reactions of 3d transition-metal complexes and metalloproteins.

## 5. Metal oxidation states with femtosecond pulses for ‘probe-before-destroy’ spectroscopy

One of the main reservations against X-ray investigations of ‘delicate’ matter such as metal complexes and proteins or molecules at XFELs is due to the concern that the unprecedented peak brilliance of XFELs damages the system to be studied. Almost 20 years ago and thus 5 years before the FLASH FEL and almost 10 years before the LCLS XFEL turned on, Neutze, Weckert and Hajdu *et al.* investigated under which circumstances meaningful structural information could be obtained from scattering of intense fs X-ray pulses such as from XFELs [83]. By simulating the Coulomb explosion of proteins, they concluded that the fs duration of the pulses helps in that the system can be probed before it is destroyed by the X-ray radiation itself. This established the ‘probe-before-destroy’ concept with fs XFEL pulses. It was experimentally verified for protein crystallography at the LCLS XFEL by the teams around Chapman, Fromme, Spence, Neutze and Schlichting [84,85] and is now an established approach often named femtosecond crystallography or serial femtosecond crystallography.

**Figure 14.**
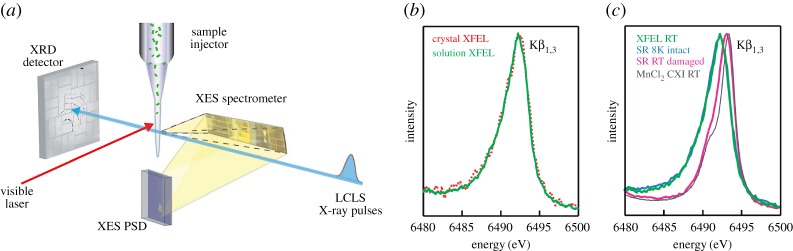
‘Probe-before-destroy’ X-ray crystallography and spectroscopy of metalloproteins with fs XFEL pulses. (*a*) Schematic depiction of the experimental set-up for simultaneous X-ray crystallography and spectroscopy of the photosystem II (PS II) protein complex at room temperature. The PS II crystal suspension is probed with fs X-ray pulses from the LCLS XFEL with X-ray diffraction (XRD detector in forward direction) and X-ray emission spectroscopy (XES spectrometer at 90° to the incident beam, a visible laser is used to illuminate the crystals and promote PS II through its reaction cycle). (*b*) Mn Kβ_1,3_ spectra of the Mn_4_CaO_5_ cluster in PS II measured at the LCLS XFEL on single crystals of PS II (red) and a solution of PS II (green, both in the so-called dark state of the system). (*c*) Mn Kβ_1,3_ spectra of the Mn_4_CaO_5_ cluster in PS II (coloured lines, dark state of PS II solution samples) and of Mn^II^ from an aqueous MnCl_2_ solution (grey line, measured at the LCLS XFEL) where the PS II spectra were measured at room temperature at the LCLS XFEL (green ‘XFEL RT’, same as the green curve in (*b*), at a synchrotron radiation source under cryogenic conditions with low X-ray dose on an intact (non-damaged) sample (blue ‘SR 8 K intact’), and at a synchrotron radiation source at room temperature where the sample was damaged (photoreduced) by the X-ray radiation (pink ‘SR RT damaged’) . Adapted with permission from [87] (Copyright © 2013 AAAS). (Online version in colour.)

Related to the topics treated here a particular complication arises for the investigation of high-valent 3d transition-metal ions in metalloproteins with XFEL radiation. The redox properties of such systems are often determined by the metal oxidation state. This makes them, in addition to being radiation-sensitive in terms of their geometric structure, prone to photoreduction where electrons created in the sample by the absorption of X-rays reduce the high-valent active metal site thereby severely altering their electronic structure [86]. This can be considered electronic-structure damage by X-rays because often the reduced metal sites are not reactive anymore and the function of the photo-reduced system is lost. Kern, Yachandra, Bergmann, Yano *et al.* recently proved [87] with simultaneous Kβ XES and X-ray diffraction of the photosystem II (PS II) protein complex at the LCLS XFEL that the fs X-ray pulses from XFELs can be used to probe high-valent metalloproteins with both intact geometric and intact electronic structures and one of the main results of this study is summarized in figure 14. The experimental approach is depicted in figure 14*a*. X-ray diffraction is used to determine the structure of PS II from crystals in a crystal suspension and Mn Kβ XES (figure 14*b,c*) is used to ascertain the high-valent states of the Mn ions in the Mn_4_CaO_5_ cluster in PS II (for the PS II sample investigated here these are two Mn ions in Mn^III^ and two in the Mn^IV^ oxidation states). The authors first found that the Mn Kβ XES spectra of the probed crystals and PS II molecules prepared in solution are identical (figure 14*b*) proving that the functional high-valent states of the Mn ions are preserved both in the crystalline and solution PS II samples. Second, as figure 14*c* shows, the solution Mn Kβ_1,3_ XES spectrum of PS II taken at room temperature at the XFEL clearly lies at lower energies compared to the Mn Kβ_1,3_ XES spectrum of a damaged PS II sample (a sample fully reduced to Mn^II^ as measured at damaging conditions at a synchrotron radiation source) and of Mn^II^ from a reference MnCl_2_ aqueous solution. Here the number of unpaired 3d electrons probed with Kβ XES is used to determine the oxidation state of the system and the lower energies in the comparison in figure 14*c* are characteristic of the high-valent Mn^III^ and Mn^IV^ states with less unpaired 3d electrons compared to Mn^II^ (figure 8*a*). This proves that the solution Mn Kβ XES spectrum of PS II from the XFEL is a measurement of intact PS II in terms of its high-valent electronic structure. It is important to note that for answering essential questions about PS II it is indispensable to study the system in its functional high-valent state in solution at room temperature [9]. Both the XFEL and synchrotron spectra in figure 14*c* (green and pink) were taken in a room-temperature solution and while they were taken at the same X-ray dose, only the XFEL measurement probes an undamaged sample in terms of electronic structure. The study by Kern *et al.* therefore basically extends the ‘probe-before-destroy’ concept from structure to electronic-structure determination and establishes the concept of ‘probe-before-destroy’ spectroscopy at XFELs.

XAS is a widely used tool to determine metal oxidation states and the case of PS II is exemplary in that it can be used to define the state-of-the-art of how to probe metal oxidation states in dilute, radiation-sensitive high-valent metalloproteins with XAS as shown in the review by Yano & Yachandra [9]. Using metal-specific XAS to probe oxidation states is tempting: One could think that the higher the oxidation state, the lower the local charge at the metal. Because with a lower charge at the metal the core-excited state would be less well screened, the energy required to perform the core to valence excitation in XAS would be higher. Accordingly, the absorption energy would be higher for higher oxidation states. This is an interpretation that in principle could apply and has in fact been applied to both K-edge and L-edge XAS of 3d transition-metal systems. Indeed, it is well established that the K-edge XAS energies [9,47,88] and the L-edge XAS energies [88,89] increase with increasing metal oxidation state (there are many more equivalently suited examples for this than in the references given here).

**Figure 15.**
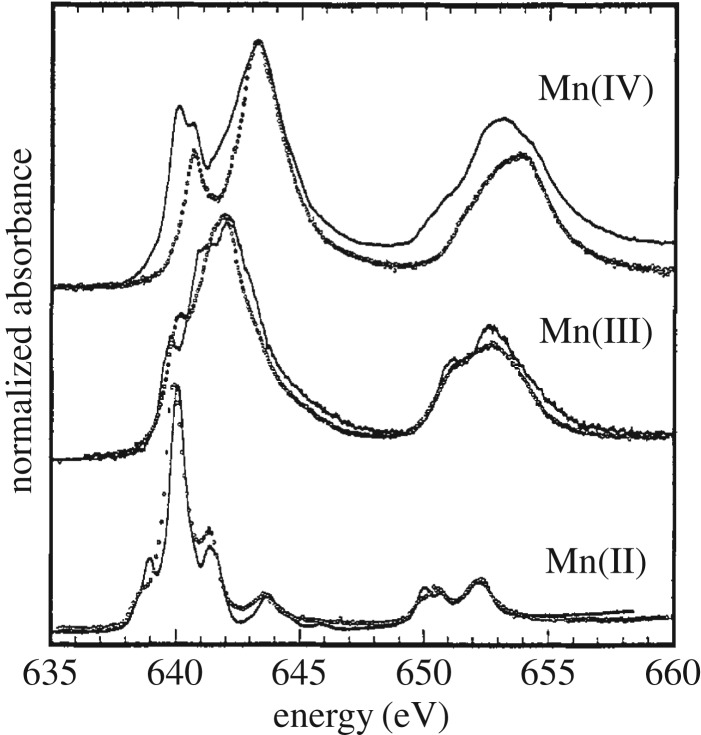
Probing the oxidation state of 3d transition-metals with metal-specific L-edge XAS. Measured Mn L-edge XAS spectra of Mn^II^, Mn^III^ and Mn^IV^ complexes (two samples for each oxidation state) establishing an average shift of the spectrum by approximately 1.5–2 eV to higher photon energies with increasing oxidation state by one. Reproduced with permission from [89]. (Copyright © 1991 American Chemical Society).

Owing to the difficulties in disentangling geometric and electronic structure effects in K-edge XAS, it is favourable to use L-edge XAS to directly probe the metal oxidation state with direct probing of the 3d-derived molecular orbitals via the strong dipole-allowed 2p → 3d transitions. Figure 15 summarizes the seminal study by Cramer, de Groot, Chen, Sette and Fuggle *et al.* on how Mn L-edge XAS energies vary with varying oxidation state [89]. This series of spectra establishes a shift of 1.5–2 eV to higher energies per unit increase in oxidation state and this shift is now commonly accepted. Besides the shift with oxidation state the spectra exhibit changes in their rich multiplet structures which can be used in addition to characterize the metal-ligand bonds [89–94].

**Figure 16.**
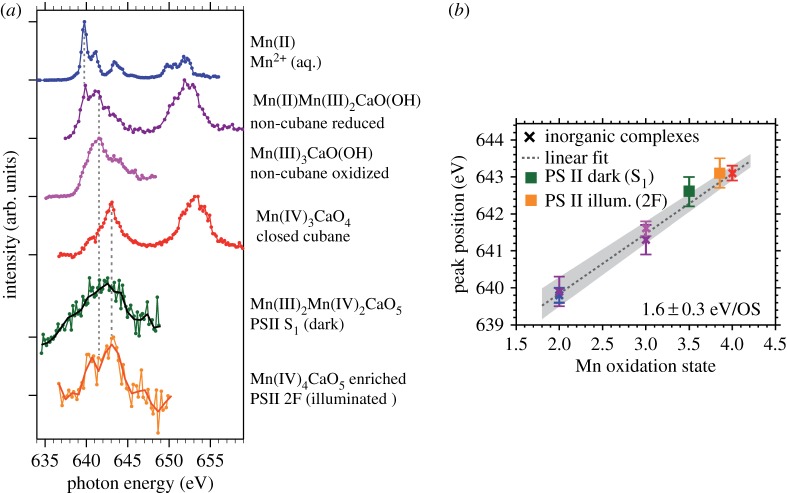
‘Probe-before-destroy’ L-edge XAS with fs X-ray pulses of high-valent 3d transition-metal complexes at room temperature and in solution. (*a*) Mn L-edge XAS spectra measured at the LCLS XFEL (in partial-fluorescence-yield, PFY, mode) of, from top to bottom, solution samples of Mn^II^ from MnCl_2_ in aqueous solution (Mn concentration 500 mM), three inorganic Mn_3_CaO_x_ complexes with different Mn oxidation states and ligand environments (Mn concentrations 6–15 mM), and the Mn_4_CaO_5_ cluster in photosystem II (PS II, Mn concentration 0.8 mM) for the S1 dark resting state (green circles, black line) and an illuminated PS II sample in an S3-enriched state (orange circles, red line). (*b*) Measured peak positions (maxima of the Mn L_3_-edge XAS) of the spectra in (*a*) as a function of Mn oxidation state (the colour code relates peak energies and spectra, note that some of the complexes contain Mn ions in different oxidation states). Reproduced with permission from [93] (licensed under a Creative Commons Attribution, CC BY, license, http://creativecommons.org/licenses/by/4.0/). (Online version in colour.)

Further concentrating on the oxidation-state shift we recently used Mn L-edge XAS to investigate high-valent Mn complexes including PS II in solution at room temperature at the LCLS XFEL and the main result is summarized in figure 16. The spectra clearly shift to higher energies with increasing Mn oxidation state (figure 16*a*). It is in particular noteworthy that the spectra of the high-valent Mn^III^ and Mn^IV^ complexes do not seem to contain any spectral contributions in the energy range characteristic of Mn^II^ (at around 639.5 eV). In addition, we observed the blue-shift of 1.5–2 eV per unit increase of oxidation state throughout the series of investigated samples (figure 16*b*). These findings led us to conclude that we successfully established ‘probe-before-destroy’ L-edge spectroscopy of high-valent 3d transition-metal systems at an XFEL and the intense fs pulses from the LCLS XFEL proved essential for this [93].

The question we were left with from this study now was: What is actually the origin of the L-edge XAS shift with oxidation state? Doubts about the interpretation that the shift is simply due to reduced screening of the core-excited state in higher oxidation states are justified because this interpretation only accounts for ground state properties (valence in the initial state of the system) and neglects effects in the final core-excited states. The situation further complicates because it turns out that quantum-chemical calculations consistently indicate that the local charge at the metal site actually does not change considerably with changing oxidation state [95,96].

**Figure 17.**
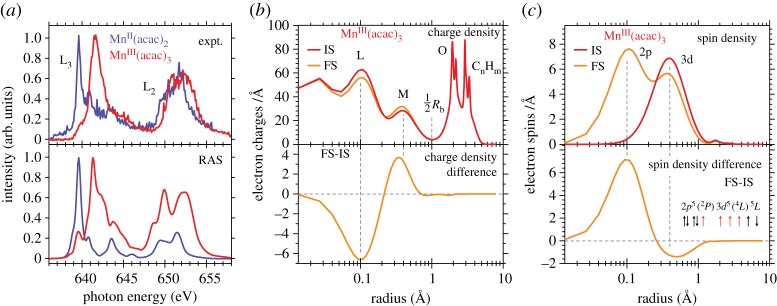
The origin of the L-edge XAS shift with oxidation state from a combination of damage-free X-ray spectroscopy and *ab initio* calculations. (*a*) Measured (top) and calculated (bottom) Mn L-edge absorption spectra (partial-fluorescence-yield, PFY, mode XAS) of Mn^II^(acac)_2_ and Mn^III^(acac)_3_ (samples measured in solution, calculations are restricted active space, RAS, calculations where the photon energies of the Mn^II^ spectrum were shifted to match the experimental spectrum at the L_3_-edge while the relative photon energies of Mn^II^ and Mn^III^ spectra are displayed as calculated, all spectra were normalized to one at maximum). (*b*) Calculated radial charge densities (RCD) of Mn^III^(acac)_3_ in the initial ground states (IS) and averaged over the final core-excited states (FS), top panel, and their difference, bottom panel (RCD of final core-excited state minus RCD of initial ground state, FS-IS). The dashed vertical lines indicate the location of L (2 s, 2p) and M (3 s, 3p, 3d) shell maxima and approximately half the Mn–O bond length *R*_b_. (*c*) Calculated radial spin densities (RSD) from RAS of Mn^III^(acac)_3_ in the IS and FS (of selected final states in the L_3_ edge only), top panel, and their difference, bottom panel (RSD of final core-excited states minus RSD of initial ground state, FS-IS). The dashed vertical lines indicate the location of the 2p and 3d shell maxima and the inset schematically depicts the dominant spin configurations in the final core-excited states (black: paired spins, red: unpaired spins, term symbols in LS-coupling. Data in (*b*) and (*c*) were extracted from the RAS calculations by placing a sphere around Mn and plotting the average electron charge (spin up plus spin down electrons) and spin densities (spin up minus spin down electrons) in this sphere versus its radius for continuously varying sphere radius. The RCD and the RSD curves are thus solid-angle integrated radial densities in electron charges and electron spins per Å. Adapted from [97] (licensed under a Creative Commons Attribution 3.0 Unported Licence, https://creativecommons.org/licenses/by/3.0/). (Online version in colour.)

To investigate this in more detail, we turned to two very simple mononuclear Mn^II^ and Mn^III^ complexes and combined damage-free L-edge XAS (at a synchrotron radiation source this time) with *ab initio* calculations of the spectra by Marcus Lundberg in a study by Kubin *et al.* which is summarized in figure 17 [97]. In the ground states of these two molecules and when going from Mn^II^ to Mn^III^ the charge population at the Mn centre changes by 0.3–0.5 electrons (depending on the method used to extract this) whereas the spin population changes by roughly one [97]. Consistent with the Kβ XES shift (figure 8*a*), the spin population (number of unpaired 3d electrons) therefore best reflects the unit change in oxidation state. Although the charge population does not change by one, the empirical correlation between oxidation state and L-edge XAS shift holds, so what is really the explanation?

Two aspects are noteworthy about the measured and calculated spectra in figure 17*a*: First, the experimental spectra are for sure free of X-ray-induced sample damage as an explicit study of such effects on these complexes showed [98]. This makes their shift amenable to a detailed investigation. Second, the theoretical L-edge XAS spectra are calculated from first principles (no adjustable parameters for the calculated relative photon energies of the two spectra). This and the good agreement with experiment enabled us to investigate the origin of the L-edge XAS shift. This became a very detailed and complex study and it is impossible to summarize all aspects here, so I restrict myself to alluding to some conceptually novel aspects. The employed theoretical approach allowed extracting the charge and spin densities in the molecules for initial ground states and for the final core-excited states and these are shown exemplarily for the Mn^III^ molecule in figure 17*b,c*. Charge density decreases in the L and increases in the M shell upon core-excitation (when going from the initial to the final states) (figure 17*b*). This directly reflects the 2p → 3d excitation with transfer of one electron (as integrated differences reveal, see [97]). The charge density in the molecule for radii above the M shell and in particular at and above half the Mn-ligand (oxygen) bond length, however, does not change upon 2p → 3d excitation. This shows that 2p-3d excitation changes charge density locally at the Mn atom only and ‘the rest of the molecule’ does not react to the excitation. Any interpretation of the L-edge XAS shift based on changes in charge densities in the metal-ligand bond and further outside of the metal centre has to, therefore, be incomplete or inappropriate. Spin density in the Mn^III^ 3d^4^ molecule upon 2p → 3d excitation increases in the 2p shell (creation of an unpaired 2p electron) and decreases in the 3d shell (effective pairing of a prior to this unpaired 3d electron) (figure 17*c*, the plotted spin densities are for selected final states in the L_3_ edge only). This surprisingly indicates that the unpaired 2p spin and the total 3d spin are parallel (the 2p and 3d spin densities are both positive, hence parallel, and the integrated differences yield that one 2p spin was created and close to one 3d spin was annihilated). This is surprising because due to the large spin-orbit interaction in the 2p shell [64,71] a predominance of parallel 2p and 3d spins for the lowest-energy states (L_3_ edge here) is not to be expected and rather one would expect an equal weight of parallel and antiparallel 2p and 3d spins (for LS-coupled states with dominant Coulomb interactions parallel orientations of core-electron and valence 3d spins are expected such as for the high-spin 3p core-hole final states of Kβ_1,3_ XES and 3p XPS, see preceding section). Based on a more detailed analysis of the calculations we found that the L-edge XAS shift reflects an increased electron affinity of Mn^III^ in the core-excited states compared to the ground state. We found that this is due to a contraction of the Mn 3d shell upon 2p → 3d excitation with concomitant changes in the direct Coulomb interactions. We could also exclude exchange Coulomb interactions and the variation of the number of unpaired 3d electrons with oxidation state as the origin for the shift.

## 6. Concluding remarks and some outlooks

X-ray spectroscopy with intense, tuneable and short-pulse X-ray radiation from XFELs can be considered a transformative tool for the investigation of 3d transition-metal complexes and metalloproteins. Concepts and insights into geometric and electronic structures can be readily transferred from steady states of chemically prepared systems to transient intermediates in photochemical reactions. This gives unique access to the nuclear dynamics and the coupled transient electronic structure in the systems. Structure, bonding and the excited state dynamics all become united by X-ray spectroscopy with fs pulses from XFELs. Quantum-chemical treatments of metal-ligand bonds are being interlaced with atomic physics and local intra-atomic multiplet effects. The necessity to understand the origins of the observed X-ray spectroscopic effects to ascertain the information content of the probe requires readdressing fundamental notions of X-ray spectroscopy. This connects classical or historic studies from some of the first X-ray sources with modern studies from the latest XFELs. Local metal oxidation states, valence orbital populations and interactions, local metal spin states, ligand-field and charge-transfer state energies, ligand coordination, bond lengths and symmetry changes, metal-ligand covalency all become accessible in transient intermediates of 3d-transition-metal complexes and in dilute radiation-sensitive metalloproteins by X-ray spectroscopy at XFELs. In combination with adequate theoretical tools such as *ab initio* calculations of electronic structures and X-ray spectra, this has the potential to reunite all these aspects. X-ray spectroscopy at XFELs allows us or may even force us to ‘go back to where the bifurcations are’, to go back to where the different aspects of the same problem got separated. Because XFELs can be used to probe different aspects of the same problem, they may enable us ‘passing by these bifurcations to bring back together’ the different aspects of the same problem. Subfields of biology, chemistry and physics get (re)united to render the unifying answer about how charge and spin densities transiently change and establish reactivity.

Some more or less obvious outlooks can be made. Time-resolved EXAFS may become available at the existing, upcoming or planed hard X-ray XFELs. Owing to their unprecedented repetition rate the upcoming LCLS-II (and the proposed LCLS-II HE where HE stands for High Energy) as well as the European XFEL will allow overcoming current limitations in sample preparation and make accessible particularly dilute species to X-ray spectroscopy. This will be essential in order to successfully extend studies from model systems to ‘real photocatalysts’ and it will enable in particular studying the local chemistry in metalloproteins. The development of time-resolved tender X-ray spectroscopy will be important to probe 4d metal systems and to ‘get the ligand view’ by probing ligand K-edges. Nonlinear versions of the X-ray spectroscopic methods discussed here will enhance selectivity to specific valence excitations at the probed metal and make accessible new observables.

**Sequel**. *I want to end by coming back to the beginning and the intention is to illustrate the role of transformative tools. For this I want to use what may be considered a revolutionary case in the history of science (without wanting to claim that the examples discussed here are revolutionary cases). My thoughts and discussions on this and on ‘how things are coming back together’ are inspired by or rather my interpretation of the work by Gérard Simon* [99] *(one should mention Roshdi Rashed as well here and his efforts in making accessible al-Haytham's work, see the corresponding reference in ref.* [99]*, and I am thankful to one of the reviewers of this contribution for pointing this out).*

*Ibn al-Haytham (Latinized Alhazen) revolutionized the field of optics around the year 1000 and his insights transformed our knowledge which was established since Euclid and Ptolemy. At the time before Ibn al-Haytham's publications it was believed that psychic emissions from the eye were ‘palpating’* [99] *objects to create vision. The so-called visual rays were thus not light rays just going in the opposite direction (from eye to object). Instead, the eye was believed to be a sensorial organ emitting ‘feeling rays’* [99]*. This is essential, as it means that optics at that time was the science of vision with a sensorial organ. The amazing thing is that at the same time mirrors for focusing light were known. But their investigation was completely unconnected from optics and vision. This in turn created troublesome contradictions and inconsistencies: If vision was based on palpating an object with a sensorial (obviously invisible) organ, how could objects appear smaller if one increased the distance to them? After all, the objects were known not to decrease in size.*

*Ibn al-Haytham now constructed a theory of vision based on light entering the eye, similar to what we know today and this transformative thinking was a revolution. His work is often illustrated with the cover picture of the Latin translation (*Opticae Thesaurus*) of Ibn al-Haytham's work on ‘Optics’* [99] *as reproduced below.*


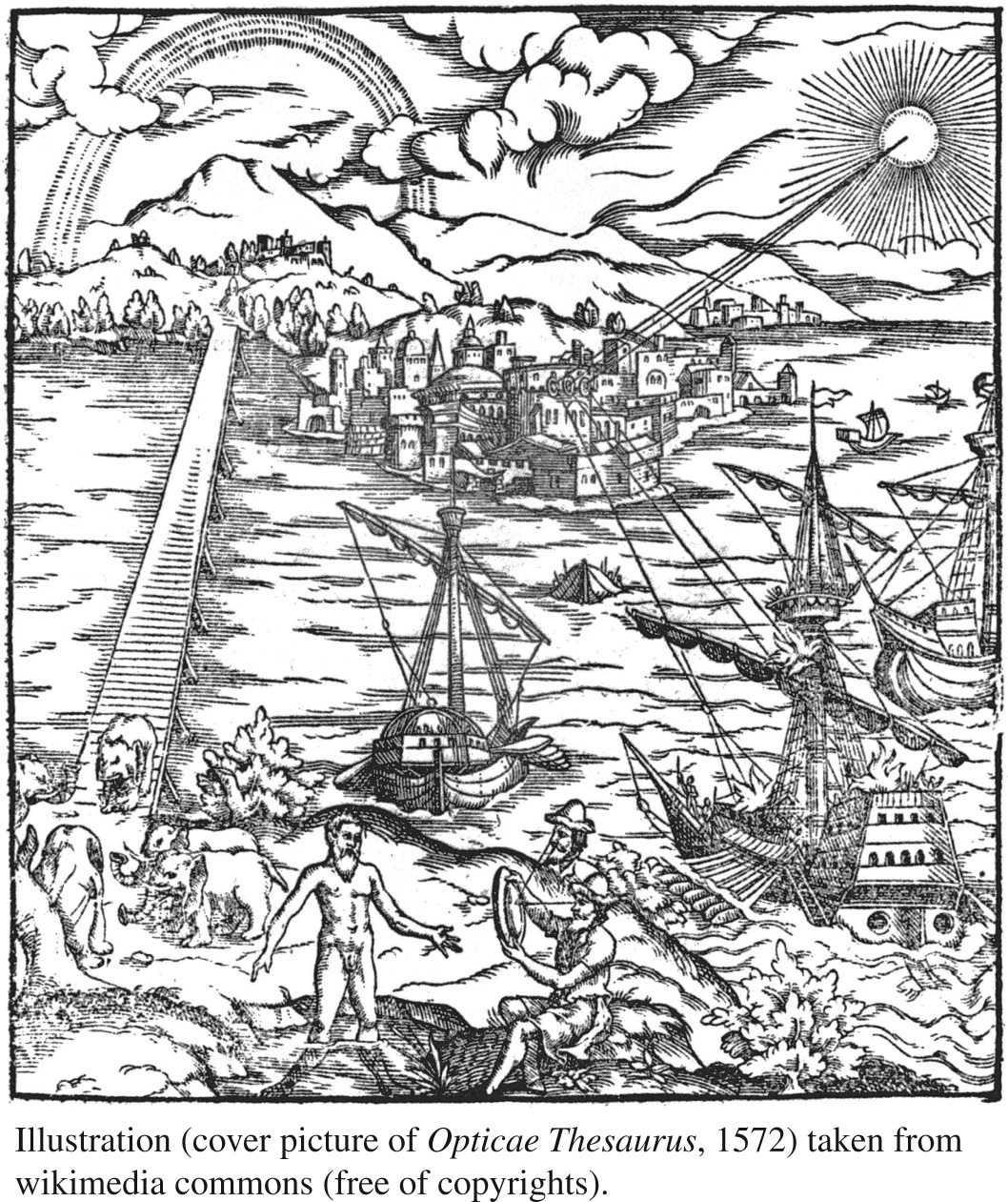


*In the context of the discussions here, the real innovation of Ibn al-Haytham's work now is not only that all the phenomena in the illustration, reflection, refraction, focusing of light, vision etc. are explained. The real innovation is that they are reunited and now all belong to one subject, optics as we know it today. So with his transformative thinking, Ibn al-Haytham ‘went back to the bifurcation’* [99] *where things were still united and his thinking brought them back together. Inconsistencies and inexplicable contradictions immediately vanished (objects do not decrease in size with increasing distance) and new developments such as the physics of light, the anatomy of vision, and the psychology of vision readily followed.*

Thinking about where the bifurcation is and how we can use transformative tools to get back there will bring things back together, bring back together different aspects of the same problem, aspects that belong together but got separated, were treated in different communities or disciplines, seemed unrelated or remained separated because they apparently contradicted each other or were inconsistent. Transformative tools deliver observables that have the potential to bring back together those aspects and to enable new explanations that relate different aspects of the same problem. In the end this will enable new insights and trigger new thoughts.

I would thus not be surprised if ‘in the laboratory on a bright day’ you found a way how to use an XFEL to ‘bring things back together’.

## Data accessibility

This article has no additional data.
